# Treatment of Primary Cervical Spine Infections: A Single-Center Analysis of the Management of 59 Patients over Three Decades

**DOI:** 10.3390/jcm14238446

**Published:** 2025-11-28

**Authors:** Myung-Jin Sung, Sung-Kyu Kim

**Affiliations:** Department of Orthopedic Surgery, Chonnam National University Medical School and Hospital, Gwangju 61469, Republic of Korea; smj2383@naver.com

**Keywords:** primary cervical infection, instrumentation, surgical debridement, antibiotics

## Abstract

**Background:** Primary cervical spine infection is a rare but rapidly progressive disease that can cause early neurological damage, leading to increased morbidity and mortality. Despite its rising incidence, optimal treatment remains controversial. This study compared clinical, hematological, microbiological, and radiological outcomes among such patients treated with different methods. **Methods:** This retrospective comparative study is a secondary analysis of a previously reported cohort of 59 patients with primary cervical spine infection between 1992 and 2018 at a single institution. Patients were stratified into conservative (Group C, *n* = 14), surgery with instrumentation (Group S + I, *n* = 32), and surgery without instrumentation (Group S, *n* = 13) groups. Outcome measures included neurological status, antibiotic duration, hematological markers, radiological parameters (segmental angle, C2–C7 angle, segmental height, fusion rate), and complications. **Results:** The mean age and follow-up period were 61.4 years and 19.4 months, respectively. Group S + I demonstrated significantly better neurological outcomes at the last follow-up (*p* = 0.047) and shorter antibiotic treatment duration (*p* < 0.001). Radiological outcomes were superior in Group S + I, with greater improvements in segmental angle (*p* < 0.001), C2-C7 angle (*p* < 0.001), mean segmental height (*p* < 0.001), and fusion rate (84.4% vs. 14.3% and 46.2% in Group C and Group S, respectively; *p* < 0.001). Group S had significantly higher complication (46.2%, *p* = 0.011) and mortality (30.8%, *p* = 0.001). Hematological and microbiological results were not significantly different among groups. **Conclusions:** Surgical debridement with anterior instrumentation provided superior outcomes compared with conservative treatment or surgery without instrumentation. Early surgery with appropriate stabilization should be considered to optimize prognosis and minimize complications.

## 1. Introduction

Primary cervical spine infection is a rare disease, accounting for only 6% of all spinal infections [1,2,3,4,5]. Cervical infection, however, is known to progress quickly and can cause neurological damage at an early stage, with increased morbidity and mortality [1,4,6]. The incidence rate of this condition is increasing due to the aging population, increasing chronic diseases that reduce immunity, and increasing non-surgical spinal treatments, such as nerve blocks [7,8]. Despite its clinical importance, research on the treatment of primary cervical infection is scarce.

Although conservative treatment with antibiotics has been preferred in the past [9,10], recent studies suggest the need for early surgical treatment. In terms of surgical methods, the use of implants in the infected operative field was traditionally contraindicated, although recent reports suggest otherwise [4,5,6]. Although treatment recommendations for pyogenic spondylodiscitis have evolved, little evidence compares conservative and surgical approaches specifically for cervical spine infection. The role of instrumentation in the infected field remains controversial, with no clear consensus on optimal treatment strategies. This study directly addresses this gap by comparing treatment outcomes. A prior study of this institution (Table S1) examined chronological trends in primary cervical spine infection across three decades [11]. In contrast, this analysis focuses on comparing treatment modalities rather than temporal patterns. While the prior investigation focused on temporal changes in patient characteristics and disease patterns, the current study addresses a distinct clinical question by comparing treatment outcomes based on management strategies (conservative treatment, surgical treatment with instrumentation, and surgical treatment without instrumentation). By analyzing the same patients through a different analytical lens, this study aimed to: (1) compare clinical outcomes among three treatment modalities, (2) evaluate radiological fusion rates and stability, and (3) provide evidence-based guidance for treatment selection in primary cervical spine infection. We hypothesized that surgical debridement combined with anterior instrumentation provides superior neurological, radiological, and functional outcomes compared to conservative or non-instrumented surgical management.

## 2. Materials and Methods

### 2.1. Patient Population

Medical records of patients who visited our hospital between 1992 and 2018 were reviewed retrospectively. Patients were included if they met the following criteria: (1) diagnosed with primary pyogenic cervical spondylodiscitis confirmed by imaging (MRI or CT) and laboratory findings (elevated inflammatory markers and/or positive cultures), (2) infection involving the cervical spine without prior spinal surgery (to exclude postoperative infections), (3) adequate clinical and radiological follow-up documentation. All cases of secondary spinal infection due to trauma, postoperative complications, or tumor invasion, as well as cases lacking radiological data, were excluded. A total of 59 patients with primary cervical spine infection were included, representing the same population described in our prior publication [11].

Both studies were conducted under the same institutional review board approval (IRB No. CNUH-2019-301). However, this study represents an independent secondary analysis with a fundamentally different research objective focused on evaluating treatment modalities and their impact on clinical, radiological, and functional outcomes, rather than examining temporal trends. The prior study stratified patients by time period (1992–2000, 2001–2009, 2010–2018), whereas the current analysis stratified the same cohort by treatment method, enabling comparative assessment of therapeutic strategies independent of temporal factors.

### 2.2. Assessments

**Figure 1:**
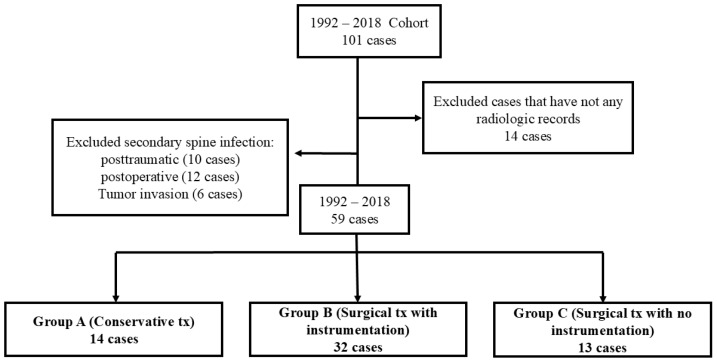
Flowchart showing patient selection and grouping.

**Figure 2:**
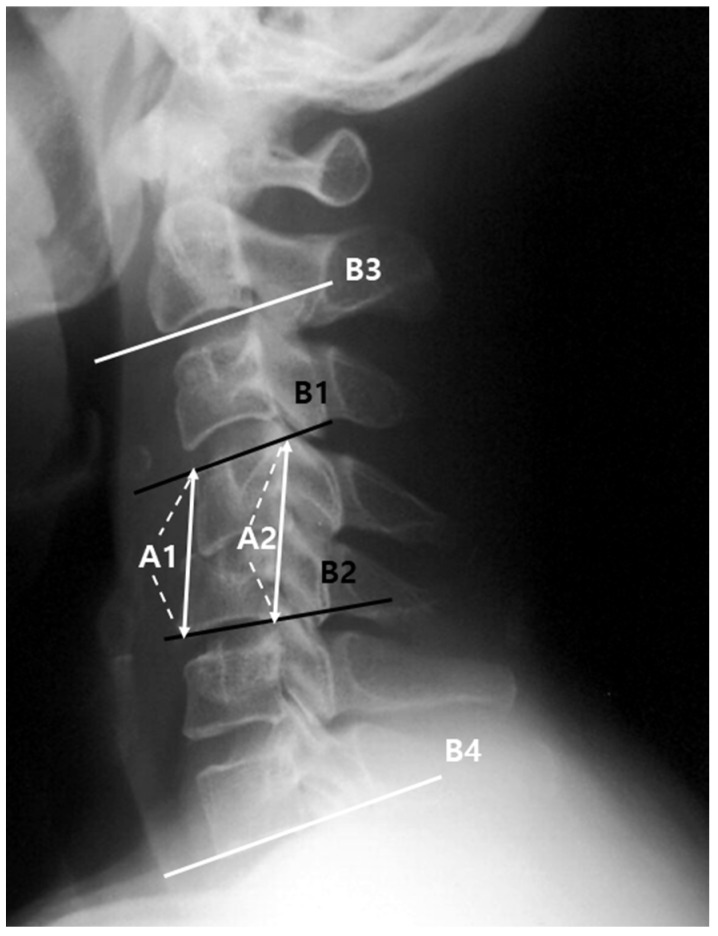
Segmental mean height: (A1 + A2)/2; Segmental angle: angle between B1 and B2; C2–C7 angle: angle between B3 and B4. A1: between the anterior margin of the upper end-plate and lower end-plate of the fused segment, A2: between the posterior margin of the upper end-plate and lower end-plate of the involved segment, B1: upper end-plate of the involved segment, B2: lower end-plate of the involved segment, B3: lower end-plate of C2, B4: lower end-plate of C7.

For the current analysis, patients were divided into three groups based on treatment approach: the conservative treatment group (Group C, *n* = 14) for those with minimal neurological deficits, no epidural abscess, or high surgical risk; the surgical treatment with instrumentation group (Group S + I, *n* = 32) for those with progressive neurological deficits, significant vertebral destruction, or epidural abscess; and the surgical treatment without instrumentation group (Group S, *n* = 13) for those with limited vertebral involvement or managed during earlier institutional practice periods (Figure 1). Blood cultures and, if possible, bone biopsies were performed for all patients. In patients who underwent surgical treatment, intraoperative microbiological biopsy and culture were performed to aid diagnosis. For clinical analysis, the American Spinal Cord Injury Association (ASIA) impairment scale was used at the first visit and at the last follow-up to assess changes in neurological symptoms. The presence of non-spinal infection, antibiotic treatment period, underlying disease, mortality, and complications of each patient were also assessed. For hematological and microbiological analyses, erythrocyte sedimentation rate (ESR), C-reactive protein (CRP) levels, and white blood cell (WBC) counts were examined, in addition to pathogen identification. For radiological analysis, simple radiographs of the cervical anteroposterior, lateral, and flexion–extension views were taken before and after treatment. To evaluate the changes according to the treatment method, the segmental angle, C2-C7 angle, mean segmental height, rate of change, and final fusion achievement were examined (Figure 2).

The rate of change in the mean segmental height was calculated as the percentage change in the mean segmental height at the last follow-up compared with that at the initial visit. Final fusion achievement was determined by a change in segmental angle of <2° on the flexion–extension radiograph at the last follow-up. Furthermore, the location of infection relative to the spinal cord (anterior; anterior epidural space, vertebral body, and intervertebral disk, and posterior; posterior epidural space, lamina, spinous process, and transverse process), the presence of epidural abscesses, and multisegmental infection were examined through contrast-enhanced magnetic resonance imaging or computed tomography in the sagittal plane.

### 2.3. Statistical Analysis

Data were analyzed using SPSS version 18.0 for Windows (IBM Corporation, Armonk, NY, USA). Continuous data are expressed as means and standard deviations, while categorical variables are expressed as percentages. The Kruskal–Wallis test was used to analyze continuous variables, and a Pearson chi-square test was performed to analyze categorical variables; for categorical variables where expected cell frequencies were less than 5, Fisher’s exact test was used instead to ensure statistical validity. A *p*-value of <0.05 was considered statistically significant.

## 3. Results

### 3.1. Demographic Analysis

The cohort comprised 42 (71%) male and 17 (29%) female patients, with a mean age of 61.4 years. The minimum outpatient follow-up period was >12 months, with an average of 19.4 months. There were 14 patients in Group C (the conservative treatment group), 32 in Group S + I (the surgical treatment with instrumentation group), and 13 in Group S (the surgical treatment without instrumentation group). For conservative treatment, antibiotics were administered orally or intravenously for 6 to 8 weeks. When the pathogen could not be identified in the blood culture, first-generation cephalosporin drugs were used as empirical antibiotics in consultation with the infectious disease department. Once the pathogen was identified, suitable antibiotics were used, considering the specific susceptibility. The duration of antibiotic use was determined in consultation with an infectious disease specialist, during which patients wore a soft collar neck brace. Indications for surgery included unbearable posterior neck pain, bacteremia, prevertebral and epidural abscesses, severe vertebral body and epiphyseal plate damage with focal kyphosis and instability, as well as neurological damage. For surgical treatment (Groups S and S + I), patients underwent surgical debridement followed by appropriate antibiotic therapy for 6 to 8 weeks (comparable to the conservative group duration). Postoperative immobilization consisted of a soft collar neck brace for 6 to 12 weeks, with duration individualized based on surgeon judgment regarding spinal stability and fusion progression in instrumented cases.

In Group S + I, all instrumentation was performed with anterior metal plate fixation along with discectomy, with three patients further subjected to fusion using a cage and allograft, and 29 subjected to fusion using an autologous iliac bone graft without a cage. In Group S, five patients underwent simple debridement alone, and eight underwent fusion using only an autologous iliac bone graft after discectomy. Male individuals accounted for the majority of patients in all groups, and there were no statistically significant differences in mean age or sex (Table 1).

### 3.2. Clinical Analysis

Table 1 summarizes the results of clinical analysis. The neurological impairment (ASIA scale grades A–D) did not significantly differ at the first visit (Group C: 64.3%, Group S + I: 40.6%, and Group S: 46.2%) (*p* = 0.153); however, a significant decrease was observed in Group S + I compared with the other groups at the last follow-up (Group C: 64.3%, Group S + I: 28.1%, and Group S: 53.8%) (95% CI: −2.1–8.5%; *p* = 0.047) (Table 2). Regarding the antibiotic treatment period, two patients in Group S + I who were treated for tuberculosis for more than 9 months were excluded from statistical analysis. The antibiotic treatment period was the shortest in Group S + I, showing a significant difference from the other groups (95% CI: 15.8–42.6; *p* < 0.001). There were no statistically significant differences among the groups in the prevalence of other underlying diseases, risk factors, and non-spinal infections accompanying the spine infection.

### 3.3. Hematological and Microbiological Analyses

Table 1 summarizes the results of hematological and microbiological analysis. The hematological analysis showed that the CRP levels and ESR were highest in Group S (CRP: 95% CI: 1.2–8.2; *p* = 0.034, ESR: 95% CI: 4.2–58.8; *p* = 0.006) at the last follow-up. Microbiological analysis identified pathogens in one (7.1%), nine (28.1%), and three (23.1%) patients in Group C, Group S + I, and Group S, respectively. The most common pathogen was methicillin-susceptible *Staphylococcus aureus*, while *Mycobacterium tuberculosis* was also identified in two patients in Group S + I. During the entire treatment period, no pathogen was identified in 46 patients (78%).

### 3.4. Radiological Analysis

**Figure 3:**
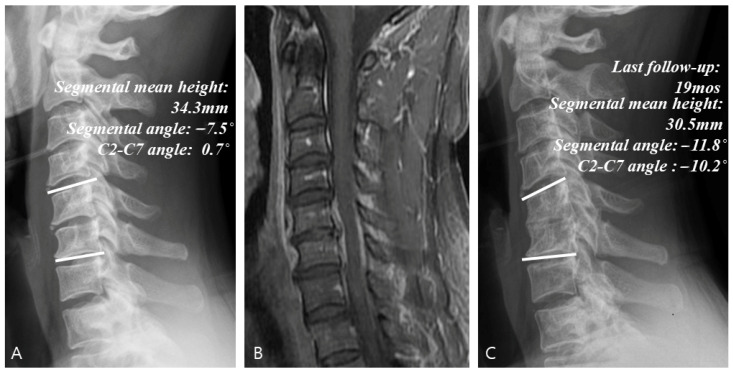
Example of a case of conservative treatment. (**A**) Simple radiographs of a 51-year-old male patient showing C5–C6 disk space narrowing and osteolytic change. (**B**) Gadolinium-enhanced MR image showing infectious spondylodiscitis at C5–C6, accompanied by prevertebral and epidural abscess. (**C**) Last follow-up (19 months) plain radiographs showing worsening radiologic parameters at C5–C6.

**Figure 4:**
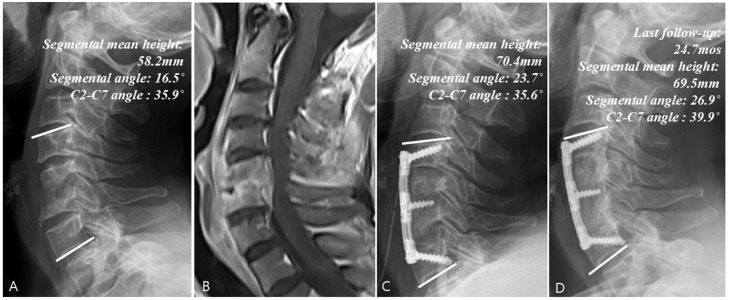
Example of a surgical treatment case. (**A**) Simple radiographs of a 50-year-old female patient showing C5–C6 vertebrae collapse. (**B**) Gadolinium-enhanced MR image showing infectious spondylodiscitis at C5–C6, accompanied by prevertebral and epidural abscess. (**C**) Immediate postoperative plain radiographs showing the state of C5–C6 corpectomy and reconstruction with plate and iliac auto bone graft. (**D**) Last follow-up (24.7 months) plain radiographs showing stable cervical spine alignment.

**Figure 5:**
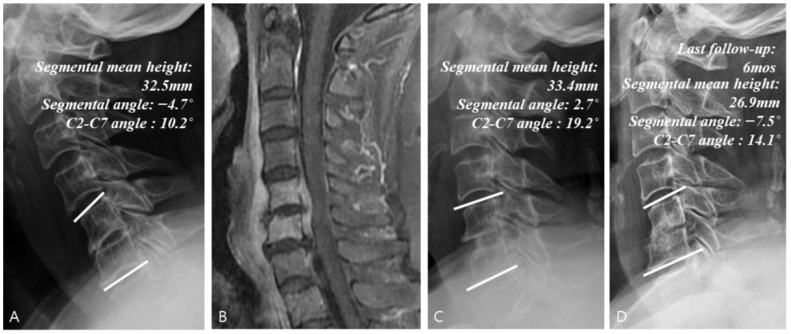
Example of a surgical treatment with no instrumentation case. (**A**) Simple radiographs of a 68-year-old female patient showing C5–C6 disk space narrowing. (**B**) Gadolinium-enhanced MR image showing infectious spondylodiscitis at C5–C6, accompanied by prevertebral and epidural abscess. (**C**) Immediate postoperative plain radiographs showing the state of C5–C6 anterior cervical discectomy and fusion with iliac auto bone graft. (**D**) Last follow-up (6 months) plain radiographs showing auto bone graft site collapse.

Table 3 summarizes the results of radiological analysis. Compared with Groups C and S, Group S + I showed significant improvements in segmental angle (95% CI: 2.8–9.2°) and C2-C7 angle (95% CI: 3.2–13.8°) at the last follow-up (*p* < 0.001). At the last follow-up, the rate of change in the mean segmental height increased significantly in Group S + I (95% CI: 18.5–42.3%; *p* < 0.001) but decreased in the other groups, while the final fusion achievement was also significantly higher in Group S + I (Group C: 14.3%, Group S + I: 84.4% and Group S: 46.2%)(95% CI: 28.4–80.2%; *p* < 0.001). Comparison of the data between the initial visit and last follow-up in Groups C (Figure 3), S + I (Figure 4), and S (Figure 5) revealed a statistically significant increase in both the segmental angle and C2–C7 angle only in Group S + I.

### 3.5. Complications

In this study, complications occurred in 12 patients, including death, and five patients who received surgical treatment underwent reoperation. Two patients in Group S (both cases of bone graft fixation failure) and three in Group S + I (two cases of screw loosening and one case of treatment failure) underwent reoperation. Deaths occurred only in Group S, with four patients dying: two died within 1 year (one at 7 months and one at 11 months) due to postoperative bacteremia, and two died approximately 2 years after surgery from diseases not related to cervical spine infection. Group S showed a significantly higher complication rate than Groups C and S + I (complications: Group C, 0%; Group S + I, 18.8%; and Group S, 46.2%) (*p* = 0.011) (Table 4). Other complications included esophageal fistula (one case), dysphagia (one case), and voice change (one case). Only one patient in Group S showed neurological impairment after surgery, with a change in the ASIA scale from E to D. Among these complications, neurological impairment was observed in the patient with bone graft fixation failure.

## 4. Discussion

This study builds upon our prior chronological analysis of primary cervical spine infection [11] by examining the same patient cohort from a treatment-based perspective. While the previous study identified temporal trends showing increased patient age, neurological impairment, and disease severity in recent years, the current analysis addresses the clinically critical question of optimal treatment selection. By stratifying patients according to management strategy rather than time period, we demonstrate that surgical debridement with instrumentation consistently produces superior outcomes regardless of when treatment occurred. This treatment-focused analysis provides complementary insights to our earlier temporal study and offers practical guidance for clinical decision-making.

Studies on primary cervical spine infection are scarce, with few cases included in these studies [1,10,11,12,13,14,15,16,17,18,19]. This study aimed to suggest an appropriate treatment method for this rare primary cervical infection by comparing the treatment outcomes according to the treatment methods over the past 30 years at this hospital.

The results of several clinical analyses (ratio of neurological impairment at last follow-up and antibiotic treatment period) and radiologic analyses (changes in the segmental angle, C2–C7 angle, mean segmental height, and final fusion achievement) suggested that treatment was most effective in the surgical treatment with instrumentation group. The use of implants, such as plates or screws, in cases of active infection has been controversial. Implants were thought to disturb antimicrobial penetration [20,21] and eradication of the infection, with an increase in septic loosening reported [22,23,24,25]. However, despite concerns regarding the presence of foreign material at the site of infection, some authors have suggested the use of implants for spinal reconstruction or stability [26,27,28,29,30,31]. There is no consensus as to which treatment method is better. While the recommended treatment method for spinal infections remains controversial, many recent studies have recommended surgical treatments, such as fusion using a metal implant at an early stage [5,32,33]. Previous studies have shown that instrumentation increases the formation of biofilms on metal and reduces the therapeutic effect [34,35]. This belief, however, is also changing, with the recent increase in the use of titanium metal implants or polyetheretherketone (PEEK) cages to reduce bacterial adhesion, resulting in greater resistance to infection [36,37]. In a study by Kim et al., which compared the surgical treatment outcomes with and without plating in 23 cases of cervical pyogenic spondylodiscitis, radiologic parameters were well-maintained in the anterior cervical discectomy and fusion with plating group until the last follow-up, with high fusion achievement [5]. There have been many reports of successful treatment for pyogenic spondylodiscitis using various implants or instrumentation. Shiban et al. obtained good results using PEEK cages and pedicle screws for pyogenic spinal infection [38], while Burkhardt et al. also obtained good results using the implant [39]. Walter et al. reported successful treatment of a patient with cervical spondylodiscitis using a PEEK cage [19]. In a study by Kim et al. conducted on a total of 134 hemodialyzed patients with pyogenic spondylodiscitis, despite the presence of more severe neurological deficit, a larger number of involved levels, and increased kyphotic angle, the instrumented surgery group showed similar treatment outcomes to the non-instrumented surgery group [40]. As shown in this study, stability achieved through thorough decompression and proper instrumentation is an important factor affecting the treatment outcome, and several other studies have also confirmed that correcting kyphosis by maintaining stability and near-normal sagittal alignment through instrumentation is more effective in treating infections [7,28,29].

The fusion achievement rate in the instrumented surgery group in our study was 84.4%, which compares favorably with the 73–89% fusion rates reported in previous studies of instrumented anterior cervical fusion for pyogenic spondylodiscitis [5,28,29]. Similarly, the complication rate of 18.8% in Group S + I in our study is consistent with the 15–25% range reported in the recent literature [38,39], while the higher complication rate of 46.2% in Group S without instrumentation highlights the importance of adequate stabilization. The mortality rates also differed between groups: 0% in Group S + I, 7.1% in Group C, and 30.8% in Group S.

Notably, Group S experienced a higher mortality rate (30.8%; 4/13) compared to 0% in both Groups C and S + I. However, this requires contextual interpretation. Of the four deaths, two occurred 2 years postoperatively from causes unrelated to spinal infection and were therefore not directly attributable to treatment differences. Excluding these, the infection-related mortality was 15.4% (2/13) in Group S versus 0% in Groups C and S + I. The higher complication rate in Group S (30.8% for infection-related complications vs. 0–18.8% in other groups) despite comparable baseline characteristics suggests treatment-related factors contributed to outcomes. Group S patients received only antibiotics without decompression or stabilization, potentially leading to residual infection and delayed healing. Elevated CRP and ESR levels at final follow-up in surviving Group S patients support this interpretation, indicating persistent inflammatory response in patients without adequate surgical intervention. These findings underscore the critical importance of timely surgical intervention, including decompression and stabilization, in the management of primary cervical spine infections.

Regarding the low microbiological yield, bacterial pathogens were isolated in only 22.0% (13/59) of patients, with 78.0% representing culture-negative cases. As we have previously documented, this finding reflects prior antibiotic administration at referring institutions that significantly reduces bacterial recovery [11]. Additionally, clinical manifestations of cervical spine infection are often nonspecific and difficult to distinguish from degenerative cervical spine disease. In such cases, diagnosis must rely on elevated inflammatory markers, characteristic MRI findings, and clinical context rather than microbiological confirmation alone. Therefore, all diagnostic and treatment decisions in this cohort were appropriately based on integrated clinical, laboratory, and radiological assessment.

### 4.1. Clinical Implications

The findings of this study have important clinical implications for the management of primary cervical spine infection. First, our results support early surgical intervention with instrumentation for patients presenting with neurological deficits, severe pain, or radiological evidence of instability or epidural abscess. Second, the significantly shorter antibiotic duration required in the instrumented surgery group (38.2 ± 8.3 days vs. 65.0 ± 14.1 days in the conservative group, *p* < 0.001) suggests that definitive surgical management may accelerate infection control and reduce prolonged antibiotic exposure and its associated risks. Third, the high complication and mortality rates observed in the non-instrumented surgery group (46.2% and 30.8%, respectively) highlight the potential risks of inadequate stabilization following surgical debridement. Clinicians should carefully weigh the risks and benefits of surgery without instrumentation, particularly in patients with compromised bone quality or significant vertebral destruction. Importantly, although treatment allocation was based on surgeon judgment rather than randomization, our baseline analysis (Table 1) demonstrates comparable demographic and clinical characteristics across groups, suggesting selection bias is unlikely. The substantial outcome disparities observed—particularly the worse outcomes in Group S (non-instrumented) compared to Group C (conservative) despite similar baseline severity—support that these differences reflect treatment efficacy rather than patient selection. Furthermore, this three-decade longitudinal study extends prior evidence by systematically comparing instrumented versus non-instrumented surgical approaches and demonstrating superior radiological (84.4% vs. 46.2% fusion rate) and clinical outcomes with instrumentation, providing robust evidence for instrumental fixation as the optimal surgical approach. Finally, our findings challenge the traditional concern about placing instrumentation in an infected field, supporting the growing body of evidence that modern titanium implants can be safely used in the setting of active infection when combined with thorough debridement and appropriate antibiotic therapy.

### 4.2. Limitations

This study has several limitations. First, we analyzed patients only from one hospital, which limits the generalizability of the conclusions to all patients with primary cervical spine infection. Additionally, although this study shares the same patient cohort with our previous chronological analysis [11], it addresses a distinct primary research question regarding the comparative effectiveness of different surgical approaches, which was not previously analyzed. This approach is scientifically justified as it provides comprehensive treatment outcome comparison from the same patient population; however, external validation through multicenter studies would strengthen the generalizability of our findings. Second, the retrospective design of this study may have introduced selection bias, as it was a non-randomized study, and the analysis relied on medical records rather than data collected prospectively. Third, the choice of treatment modality was based on the surgeon’s judgment rather than standardized criteria, which introduced inherent selection bias. Although baseline demographic and clinical characteristics were comparable across groups (Table 1), residual confounding from unmeasured disease severity factors cannot be entirely excluded. Fourth, the relatively small sample size, particularly in Groups C (*n* = 14) and S (*n* = 13), may have limited statistical power to detect differences in some outcome measures.

Despite these limitations, our findings provide valuable evidence supporting surgical debridement with instrumentation as the optimal treatment for primary cervical spine infection. Future research should focus on prospective multicenter studies with standardized treatment protocols and long-term follow-up to further validate these findings.

## 5. Conclusions

Within the limitations of this retrospective single-center study, surgical debridement with instrumentation appears to provide superior outcomes compared with conservative or non-instrumented surgical treatment for primary cervical spine infection. Thorough surgical debridement coupled with stabilization via instrumentation demonstrated favorable clinical and radiological outcomes. While acknowledging the inherent limitations of our retrospective design, these findings suggest that early surgical intervention incorporating appropriate instrumentation may optimize patient outcomes and reduce complications in appropriately selected patients with primary cervical spine infection.

## Figures and Tables

**Table 1:** Clinical, hematological, and microbiological analyses.

Parameter	Total (*n* = 59)	Group C (*n* = 14)	Group S + I (*n* = 32)	Group S (*n* = 13)	*p*-Value	95% CI
Mean age (Year)	61.4 ± 10.0	62.5 ± 10.6	60.0 ± 9.6	63.8 ± 10.4	0.464 *	(−1.8, 2.4)
Sex (Male/Female)	42/17	13/1	20/12	9/4	0.153 ^†^	-
Antibiotic treatment duration (Day)	47.4 ± 15.3 (*n* = 57) **	65.0 ± 14.1	38.2 ± 8.3 (*n* = 30) **	49.5 ± 11.5	0.033 *	(15.8, 42.6)
Non-spinal infection	10	3	4	3	0.558 ^†^	-
Underlying disease and risk factor	30	6	15	9	0.314 ^‡^	-
WBC count (µL)						
-first visit	10,333.1 ± 4318.9	11,071.4 ± 6526.3	9857.8 ± 3500.7	10,707.7 ± 3311.5	0.729 *	(−1500, 2200)
-last follow-up	7027.1 ± 3504.1	7007.1 ± 3484.4	6231.3 ± 2485.4	9007.7 ± 4929.4	0.138 *	(−800, 2800)
CRP level (mg/L)						
-first visit	8.8 ± 8.1	10.2 ± 7.8	6.7 ± 7.2	12.5 ± 9.3	0.062 *	(−2.1, 1.5)
-last follow-up	2.2 ± 4.4	1.5 ± 3.3	1.4 ± 1.5	5.0 ± 8.0	0.034 *	(1.2, 8.2)
ESR (mm/h)						
-first visit	80.9 ± 30.8	80.2 ± 28.3	79.7 ± 28.5	84.6 ± 40.0	0.636 *	(−12.5, 10.8)
-last follow-up	40.0 ± 30.1	21.3 ± 21.0	41.8 ± 25.9	55.5 ± 38.8	0.006 *	(4.2, 58.8)
Pathogen	13/59	1/14	9/32	3/13	0.286 ^‡^	-
-MRSA	1	0	0	1		
-MSSA	7	1	4	2		
-Gram-negative	1	0	1	0		
-others	4	0	4	0		

Continuous variables are expressed as means (standard deviations). WBC, white blood cell; CRP, C-reactive protein; CI, confidence interval; ESR, erythrocyte sedimentation rate; MRSA, Methicillin-resistant *Staphylococcus aureus*; MSSA, Methicillin-susceptible *Staphylococcus aureus*. * Kruskal–Wallis test, ^‡^ Pearson’s chi-square test, ^†^ Fisher’s exact test. ** Exclusion of two patients due to tuberculosis.

**Table 2:** Neurologic symptom analysis.

ASIA Impairment Scale (First Visit → Last Follow-Up)	Group C (*n* = 14)	Group S + I (*n* = 32)	Group S (*n* = 13)	*p*-Value	95% CI
Neurologic impairment					
-First visit	10	13	6	0.153 ^‡^	-
-Last follow-up	9	9	7	0.047 ^‡^	(−2.1%, 8.5%)
Grade A	0 → 0	0 → 0	0 → 0		
Grade B	1 → 1	1 → 0	2 → 1		
Grade C	0 → 0	2 → 1	3 → 0		
Grade D	9 → 8	10 → 8	1 → 6		
Grade E	4 → 5	19 → 23	7 → 6		

^‡^ Pearson’s chi-square test. ASIA, the American Spinal Cord Injury Association.

**Table 3:** Radiological analysis.

Parameter	Group C (*n* = 14)	Group S + I (*n* = 32)	Group S (*n* = 13)	*p*-Value	95% CI
Epidural abscess	5	13	6	0.859 ^‡^	-
Location of infection					
-Anterior	13	30	13	0.635 ^‡^	-
-Posterior	1	1	0		
-Anteroposterior	0	1	0		
Multisegmental Infection	3	13	4	0.353 ^†^	-
Segmental angle (°)					
-First visit	−2.7 ± 3.1	−2.4 ± 5.9	−2.7 ± 4.7	1.000 *	(−1.2, 1.5)
-Last follow-up	−3.1 ± 3.8	2.7 ± 5.4	−2.8 ± 4.6	<0.001 *	(2.8, 9.2)
*p*-value ^a^	0.724 ^	<0.001 ^	0.981 ^		
C2-C7 angle (°)					
-First visit	8.4 ± 8.1	8.2 ± 7.8	9.4 ± 6.0	0.797 *	(−2.1, 2.5)
-Last follow-up	8.1 ± 6.8	16.7 ± 8.4	9.1 ± 7.3	0.001 *	(3.2, 13.8)
*p*-value ^a^	0.859 ^	<0.001 ^	0.744 ^		
Mean height change (%)	−19.3 ± 11.9	14.0 ± 15.8	−7.8 ± 10.9	<0.001 *	(18.5, 42.3)
Fusion achievement	2/14	27/32	6/13	<0.001 ^‡^	(28.4%, 80.2%)

*p*-value ^a^: comparison between the first visit and last follow-up. * Kruskal–Wallis test, ^‡^ Pearson’s chi-square test, ^†^ Fisher’s exact test, ^ Paired *t*-test. Location of infection: anterior = anterior epidural space or vertebral body or intervertebral disk, posterior = posterior epidural space or lamina or spinous process or transverse process. Angle (°): positive value = lordosis, negative value = kyphosis

**Table 4:** Complications.

Parameters	Group C (*n* = 14)	Group S + I (*n* = 32)	Group S (*n* = 13)	*p*-Value	95% CI
Total complications	0/14	6/32	6/13	0.021 ^†^	(8.5%, 52.1%)
-Reoperation	0	3	2		
-Esophageal fistula	0	1	0		
-Dysphagia	0	1	0		
-Voice change	0	1	0		
-Neurologic impairment	0	0	1		
-Death	0	0	4	0.001 ^†^	(1.2%, 18.5%)

^†^ Fisher’s exact test

## Data Availability

Data were not publicly available due to ethical reasons and patient privacy.

## Supplementary Materials

The following supporting information can be downloaded at https://www.mdpi.com/article/10.3390/jcm14238446/s1, Table S1: Prior study comparative.
