# Supplementary material for: Treatment of Primary Cervical Spine Infections: A Single-Center Analysis of the Management of 59 Patients over Three Decades
Source: J Clin Med. 2025 Nov 28;14(23):8446. doi: 10.3390/jcm14238446 (PMC12693083; doi:10.3390/jcm14238446)
Supplement: Supplementary file 1 [file jcm-14-08446-s001.zip › jcm-3984852-supplementary.pdf]

Table S1. Prior study comparative.

Aspect	Prior Study (2022)	Current Study (2025)
Study Population	59 patients (1992-2018)	Same 59 patients (1992-2018)
Stratification Method	Time-Based (3 groups)	Treatment-Based (3 groups)_
Group Division	Group A: 1992-2000 (n=12)<br>Group B: 2001-2009 (n=26)<br>Group C: 2010-2018 (n=21)	Group C: Conservative (n=14)<br>Group S+I: Surgery + Instrumentation (n=32)<br>Group S: Surgery only (n=13)
Primary Comparison	Disease patterns across decades	Treatment outcomes across modalities
Main Finding	Increasing severity over time	Instrumented surgery superior
Clinical Question	How has disease changed?	Which treatment optimized outcomes?
Impact	Epidemiological trends	Treatment selection guidance
